# Pulmonary vein signal in mitral regurgitation

**DOI:** 10.1186/s13054-018-2031-z

**Published:** 2018-05-11

**Authors:** Tim Balthazar, Bart Jacobs, Jens-Uwe Voigt

**Affiliations:** Department of cardiology, University Hospitals Leuven, Herestraat 49, 3000 Leuven, Belgium

We read the article Critical care ultrasonography in acute respiratory failure [1] by Vignon et al. with great interest. We agree that echocardiography plays an important role in the evaluation of patients with acute respiratory failure. We do not, however, agree with the statement that massive mitral regurgitation causes a reversal in pulmonary vein diastolic inflow or “D wave” as implied in Figure 2 where a case of cardiac weaning failure is presented. Since mitral regurgitation is a systolic phenomenon it can only cause reversal of the systolic “S wave” [2]. This is actually shown in the right lower panel of the figure, where the arrow points at a reversed “S wave”. The “S” and “D” labels should be exchanged and the figure legend should be corrected accordingly.
