# Supplementary material for: Pulmonary vein signal in mitral regurgitation
Source: Crit Care. 2018 May 11;22:123. doi: 10.1186/s13054-018-2031-z (PMC5946452; doi:10.1186/s13054-018-2031-z)
Supplement: Related texts

# Reply: Authors’ response

**Authors:** Philippe Vignon, Xavier Repessé, Antoine Vieillard-Baron, Eric Maury

We thank Dr. Balthazar and coworkers for pointing out the erroneous figure legend and annotation in our recently published manuscript describing the clinical value of critical care ultrasonography in both the diagnostic work-up and management of patients admitted to the intensive care unit with acute respiratory failure [1]. Massive mitral regurgitation results in an extended reversal flow which reaches the left atrial posterior wall and regurgitates within pulmonary veins during systole. Accordingly, as adequately emphasized by the authors, the systolic component of the pulmonary vein Doppler pattern (S wave) is either blurred or even inverted, whereas the diastolic component (D wave) is markedly prominent due to associated elevated left ventricular filling pressure. During systole, S wave blurring or reversal has the same diagnostic value as the presence of a markedly elevated “v” wave on the pulmonary capillary wedge pressure curve during right heart catheterization [3]. In this clinical setting, the predominant pulmonary vein Doppler D wave is usually associated with a predominant mitral Doppler E wave typically consistent with a normalized or even restrictive mitral Doppler pattern. These Doppler profiles reflect both the volume overload and associated increased filling pressures secondary to the massive mitral regurgitation. Of note, Doppler S wave blurring or reversal may be observed predominantly in left or right pulmonary veins when the mitral regurgitant jet is eccentric and has either a clockwise or anticlockwise propagation along left atrial walls, respectively [4].
